# GFPT2 expression is induced by gemcitabine administration and enhances invasion by activating the hexosamine biosynthetic pathway in pancreatic cancer

**DOI:** 10.1007/s10585-024-10298-y

**Published:** 2024-06-18

**Authors:** Kent Miyazaki, Kyohei Ariake, Satoko Sato, Takayuki Miura, Jingyu Xun, Daisuke Douchi, Masaharu Ishida, Hideo Ohtsuka, Masamichi Mizuma, Kei Nakagawa, Takashi Kamei, Michiaki Unno

**Affiliations:** 1 Department of Surgery, Tohoku University Graduate School of Medicine, Sendai, Japan; 2 Department of Gastroenterological Surgery, Sendai City Medical Center Sendai Open Hospital, Sendai, Japan; 3 Department of Pathology, Tohoku University Hospital, Sendai, Japan

**Keywords:** Cancer, Chemotherapy-induced metastasis, Hexosamine biosynthesis pathway, Glutamine-fructose-6-phosphate transaminase 2, Neoadjuvant chemotherapy

## Abstract

Our previous studies revealed a novel link between gemcitabine (GEM) chemotherapy and elevated glutamine-fructose-6-phosphate transaminase 2 (GFPT2) expression in pancreatic cancer (PaCa) cells. GFPT2 is a rate-limiting enzyme in the hexosamine biosynthesis pathway (HBP). HBP can enhance metastatic potential by regulating epithelial-mesenchymal transition (EMT). The aim of this study was to further evaluate the effect of chemotherapy-induced GFPT2 expression on metastatic potential. GFPT2 expression was evaluated in a mouse xenograft model following GEM exposure and in clinical specimens of patients after chemotherapy using immunohistochemical analysis. The roles of GFPT2 in HBP activation, downstream pathways, and cellular functions in PaCa cells with regulated GFPT2 expression were investigated. GEM exposure increased GFPT2 expression in tumors resected from a mouse xenograft model and in patients treated with neoadjuvant chemotherapy (NAC). GFPT2 expression was correlated with post-operative liver metastasis after NAC. Its expression activated the HBP, promoting migration and invasion. Treatment with HBP inhibitors reversed these effects. Additionally, GFPT2 upregulated ZEB1 and vimentin expression and downregulated E-cadherin expression. GEM induction upregulated GFPT2 expression. Elevated GFPT2 levels promoted invasion by activating the HBP, suggesting the potential role of this mechanism in promoting chemotherapy-induced metastasis.

**Supplementary Information:**

The online version contains supplementary material available at 10.1007/s10585-024-10298-y.

## Introduction

Pancreatic cancer (PaCa) is an intractable form of gastrointestinal cancer [1, 2]. According to World Health Organization (WHO) statistics, its 5-year survival rate is only 9%. The incidence of PaCa has been increasing, with an average annual increase of 1.03% between 1971 and 2014 in the United States [3]. The prevalence of PaCa has also increased in Japan [4]; in 2018, 42,359 patients were newly diagnosed with PaCa, and the total number of deaths was 36,356 in 2019 [4]. Thus, most patients die of PaCa despite improvements in the treatment in recent years.

Chemotherapy plays an indispensable role in PaCa treatment; most patients with unresectable PaCa receive chemotherapy [5]. Patients eligible for resection are also recommended to receive preoperative or postoperative adjuvant chemotherapy [6]. However, continuous administration of anticancer drugs causes resistance and leads to disease progression, including tumor regrowth or the appearance of new metastatic lesions. Recently, chemotherapy-induced metastasis (CIM) has been emerged as a new critical problem [6]. In CIM, chemotherapy achieves an unfavorable outcome contrary to the intended effect; tumor growth is suppressed, as expected; however, metastatic potential is promoted [7]. When repairing the damage caused by chemotherapy, mutations accumulate in cancer cells. This sometimes leads to epithelial-mesenchymal transition (EMT) and the promotion of metastasis [8]. Chemotherapy also changes the tumor microenvironment by releasing inflammatory cytokines or chemokines that activate immune cells, thereby forming a suitable environment for metastasis [6, 9–14]. However, the detailed mechanisms of the action of CIM in PaCa remain unclear.

Gemcitabine (GEM), the gold standard drug for treating PaCa [5], functions by targeting cancer cell DNA and inhibiting its synthesis, suppressing cancer cell division and growth. GEM increases intracellular reactive oxygen species (ROS) levels and damages mitochondria, inducing cancer cell apoptosis [15]. Our previous studies have demonstrated that GEM increases the expression of stomatin-like protein 2 (SLP2), a mitochondrial membrane protein that crucially promotes metastasis [16]. SLP2 expression is elevated in patients after preoperative chemotherapy [16]. Microarray analysis has revealed a strong correlation between glutamine-fructose-6-phosphate transaminase 2 (GFPT2) and SLP2. Additionally, GFPT2 expression was elevated in PaCa cells after GEM exposure, suggesting that, as with SLP2, GFPT2 promotes metastasis after chemotherapy.

GFPT2 is a subtype of GFPT1 with 75.6% amino acid homology [17]. Both GFPTs are rate-limiting enzymes in the hexosamine biosynthetic pathway (HBP). HBP is a branch of the glucose metabolism pathway [18] by which 2–5% of glucose is metabolized [19]. The HBP pathway begins with the GFPT-catalyzed formation of glucosamine-6-phosphate formation from glutamine and fructose-6-phosphate (a glycolytic metabolite) as substrates. Subsequent reactions, catalyzed by metabolic enzymes, ultimately produce UDP-N-acetylglucosamine (UDP-GlcNAc). Glycosylation, a post-transcriptional modification vital for various cellular functions [20], is achieved by the addition of O-N-acetylglucosamine (O-GlcNAc) from the final products of the HBP, to serine and threonine residues by glycosyltransferase. In cancer cells, β-catenin [21], c-Myc [22], and NF-κB [23] are glycosylated to promote their activation. These factors promote migration and invasion, lead to EMT, or induce resistance to anticancer drugs [24]. Therefore, if GFPT2 expression is elevated after initiating chemotherapy, HBP activation may play a crucial role in promoting metastasis.

In PaCa, GFPT1 promotes aggressiveness by activating HBP, while high GFPT1 expression predicts poor prognosis [25, 26]. However, GFPT2 expression has not been well characterized, and no studies have been conducted on GFPT2 in PaCa. On the basis of these findings, the GEM stimulation-mediated increase in GFPT2 expression led us to hypothesize that chemotherapy promotes the metastatic potential of PaCa via the HBP. Hence, the aim of this study was to evaluate whether GEM treatment increases GFPT2 expression in vivo and whether increased GFPT2 expression promotes metastatic potential by activating HBP.

## Materials and methods

### Antibodies, reagents, and cell lines

The PaCa-2, BxPC3, and PK1 cell lines were obtained from the CELL BANK of the RIKEN Bio Resource Center (Tsukuba, Japan), whereas the PANC-1 cell line was purchased from the American Type Culture Collection (Manassas, VA, USA). The cells were cultured in RPMI 1640 medium (Sigma Aldrich, St. Louis, MO, USA) supplemented with fetal bovine serum (FBS) (10%; Sigma Aldrich) and penicillin/streptomycin (1%; Thermo Fisher Scientific, Inc., Waltham, MA, USA) at 37 °C in a 5% CO_2_ environment. Gemcitabine hydrochloride (FUJI FILM WAKO, Osaka, Japan) and 6-diazo-5-oxo-L-norleucine (DON) (D2141; Merck, Lebanon, NJ, USA) were dissolved in the cell culture medium at the indicated concentrations immediately before use.

Antibodies against GFPT2 were obtained from Abcam (cat: ab190966; Cambridge, UK); O-GlcNAc from Merck (cat: MABS157); TCF8/ZEB1 from Cell Signaling Technology (CST) (cat: 3396; Danvers, MA, USA); E-cadherin from CST (cat: 3195); vimentin from CST (cat: 5741); and β-actin from Proteintech (cat: 20536-I-AP; Rosemont, IL, USA). Anti-mouse IgG (cat: 7076) and anti-rabbit IgG (cat: 7074) secondary antibodies were both purchased from CST.

### Cloning of cells stably suppressed by GFPT2

The nucleotide sequences corresponding to *GFPT2* shRNAs are detailed in Online Resource 1. These shRNAs were incorporated into the pBAsi-hU6 NEO plasmid (Code. no. 3227; Takara Bio, Kusatsu, Japan). Lipofectamine 2000 reagent (Cat. no. 11668019; Thermo Fisher Scientific) was used for plasmid transfection, following the manufacturer’s protocol. As a negative control, an empty pBAsi-hU6 NEO vector was transfected into SUIT2 cells. Clones that underwent transfection were subjected to selection on RPMI medium supplemented with 800 mg/mL Geneticin® (cat: 10131–027; Sigma Aldrich) for 3 weeks. Subsequently, a single colony was selected for cultivation.

### Cloning of cells stably overexpressing GFPT2

The *GFPT2* expression vector (cat: RC200519) was obtained from OriGene Technology (Rockville, MD, USA). Lipofectamine 2000 was used to transfect the vector into PANC-1 cells, following the manufacturer’s protocol. In addition, a blank pCMV6-Entry vector (cat: PS100001; OriGene Technology) was transfected into PANC-1 cells as a negative control. Transfected cells were selected on RPMI medium supplemented with 800 mg/mL Geneticin® after 3 weeks of incubation, after which a single colony was picked and cultured.

### Quantitative real-time reverse transcription polymerase chain reaction (q-PCR)

RNA was extracted from cells using a Nucleospin RNA Kit (cat: 740,955; Takara Bio). Reverse transcription was conducted using the PrimeScript RT Master Mix (cat: RR036A; Takara Bio) following the manufacturer’s protocol. The thermocycling procedure was 37 °C for 15 min, followed by 85 °C for 5s. The resulting products served as templates for RT-PCR and were subjected to amplification using TB Green Premix Ex Taq II and ROX Plus (Cat: RR82LR; Takara Bio), with the following thermocycling procedure: 95 °C for 30s; 40 cycles of denaturation at 95 °C for 5s, annealing/extension at 60 °C for 30 s; and annealing/extension at 60 °C for 30s. The expression levels of the genes in each sample were determined according to the 2^−ΔΔCq^ method [27]. Relative quantities were analyzed after normalization to β-actin expression levels. The primer sequences are listed in Online Resource 1. All in vitro experiments were conducted in biological triplicate.

### Western blotting (WB)

Cell lysis was performed using radioimmunoprecipitation assay (RIPA) buffer (Thermo Fisher Scientific), and protein concentrations were calculated using a BCA kit (Thermo Fisher Scientific). Subsequently, 20 mg of protein was separated via SDS-PAGE (4–15%) and transferred onto PVDF membranes (Bio-Rad, Hercules, CA, USA). The membranes were subsequently blocked with 0.5% TBS-T supplemented with 5% skim milk and incubated with primary antibodies at a 1:2000 dilution. The sections were then incubated with horseradish peroxidase (HRP)-conjugated secondary antibodies at a 1:3000 dilution. Protein signals were analyzed using Clarity ECL Western Substrate (cat: 1705062; Bio-Rad). Bands were visualized using an ImageQuant LAS 4000 mini system (GE Healthcare, Buckinghamshire, UK). The relative quantities were analyzed after normalization of the internal control to β-actin expression.

### Cell proliferation assay

A total of 5.0 × 10^3^ cells were passaged into 96-well plates and cultured in 100 μL of medium supplemented with 10% FBS and 1% penicillin. MTS assays were conducted using the CellTiter 96-well assay reagent (cat: G358B; Promega, Madison, WI, USA) according to the manufacturer’s protocol.

### Cytotoxicity assay

A total of 5.0 × 10^3^ cells were seeded into 96-well plates. After 24 h, the medium was replaced with gemcitabine-containing medium at concentrations ranging from 1 × 10^–3^ to 1 × 10^3^ µM. After 120 h of culture, cell viability was assessed using the CellTiter 96-well assay reagent and the MTS assay.

### Wound healing assay

The cells were cultured in six-well plates to 80% confluence and the medium was changed for serum starvation. After 24 h, the wound was scratched with a sterile 10 μL pipette tip. Next, the cells were rinsed with PBS and cultured in a medium supplemented with 10% FBS and 1% penicillin. The extent of wound closure was measured after 18 h and 24 h for SUIT2 and PANC-1 cells, respectively, at three time points per group; subsequently, the average distance was calculated. The calculated data were presented as relative indices based on the initial scratch length.

### Invasion assay

A Boyden chamber (cat: ECM554; Millipore) coated with a layer of ECMatrix™ was used for the invasion assays. Cells were serum-starved for 24 h and subsequently harvested with serum-free medium. Next, 300 μL (1.0 × 10^6^/mL) of cells was added to the upper chamber, and 500 L of 10% FBS-containing medium with or without the HBP inhibitor was added to the lower chamber. After 24 h, the invading cells were detached from the underside of the upper chamber using 225 µL of cell detachment solution at 37 °C for 30 min. Subsequently, the detached cells were stained with 75 μL of lysis buffer/dye solution at 20–25 °C for 15 min and quantified using a fluorescent plate reader equipped with a 480/520 nm filter. Data are presented as a relative index determined by setting the percentage of invading control cells to 100%.

### Mouse xenograft model

The study protocol was approved by the Institutional Animal Experiment Committee of Tohoku University (Sendai, Japan) on September 29, 2021. An orthotopic transplant model was established according to the experimental protocol outlined by Sugisawa et al. [11]. PANC-1 cells (2 × 10^6^ cells, 0.2 mL) were lysed in PBS and subcutaneously injected into the shoulders of nude mice. Subsequently, the developed tumors were excised and fragmented into 5 mm^3^ pieces. A triple-mixed anesthetic solution comprising midazolam (5 mg/mL), butorphanol (5 mg/mL), and medetomidine (1 mg/mL) was injected intraperitoneally at 0.1 mL per 10 g body weight. A 10 mm transverse incision was made in the left flank of the mouse, and the pancreatic tail was exposed. Tumor fragments were orthotopically implanted into the pancreatic tail using a 7–0 nylon suture (BEAR Medic Corporation, Kuji-gun, Japan). The incision was closed using a 5–0 nylon suture (BEAR Medic Corporation). After the tumor volume reached 60 mm^3^ in the pancreatic tail, the animals were divided into three groups: group 1, the untreated control group; group 2, low-dose GEM (25 mg/kg/week); and group 3, high-dose GEM (125 mg/kg/week). GEM was administered once a week for 6 weeks [11]. One week after the last treatment, the tumors formed were excised and homogenized. The protein expression was determined via WB.

### Immunohistochemistry

All specimens used for immunohistochemistry were fixed in 10% formalin for 24 h and embedded in paraffin wax. GFPT2 immunostaining was performed by a pathology specialist who also scored immunoreactivity. A semi-quantitative analysis of immunoreactivity was performed by combining the staining intensity with the percentage of positive cells. The staining intensity was graded as 0 (negative), 1 (weak), 2 (moderate), or 3 (strong) (Online Resource 2), whereas the percentage of positive cells was scored as 0 (0–5%), 1 (6–25%), 2 (26–50%), 3 (51–75%), or 4 (> 76%). The immunoreactive score (IRS), ranging from 0 to 12, was determined by multiplying the staining intensity by the percentage of positive cells.

Clinical data of 161 patients diagnosed with pancreatic ductal adenocarcinoma who underwent pancreatectomy between 2012 and 2016 were obtained from the clinical and pathological records maintained at Tohoku University on January 1, 2022. Resectability was defined according to the National Comprehensive Cancer Network guidelines [5], and pathological diagnosis was based on the Classification of Pancreatic Carcinoma (fourth English edition) [28]. The Institutional Review Board of Tohoku University (Sendai, Japan) approved the study design on September 29, 2021 (2021-1-591). As this was a retrospective cohort study, the opt-out method was used instead of obtaining informed consent.

### Gene expression analysis

Correlations between GFPT2 and EMT-related factors were analyzed using the Gene Expression Profiling Interactive Analysis (GEPIA), an analysis platform leveraging The Cancer Genome Atlas (TCGA) and the Genotype-Tissue Expression (GTEx) databases, to analyze the downstream signaling pathways implicated in the HBP activated by GFPT2 [29–31].

### Statistical analysis

Statistical analyses were performed using JMP Pro software version 16 (SAS Institute, Cary, NC, USA). For the analysis of clinical specimens, continuous variable comparisons were performed using the Kruskal‒Wallis test, and group comparisons were assessed using Pearson’s chi-square test. The experimental data are presented as the mean ± standard deviation (SD), and Student’s *t*-test was used to compare the two groups. Survival rates were calculated using the Kaplan–Meier method, and group comparisons were conducted using the log-rank test. Overall survival or recurrence-free survival was calculated from the date of surgical resection to the date of death, recurrence, or censoring. Statistical significance was set at P < 0.05.

## Results

### Establishment of GFPT2-suppressed cells and GFPT2-overexpressing cells

**Fig. 1:**
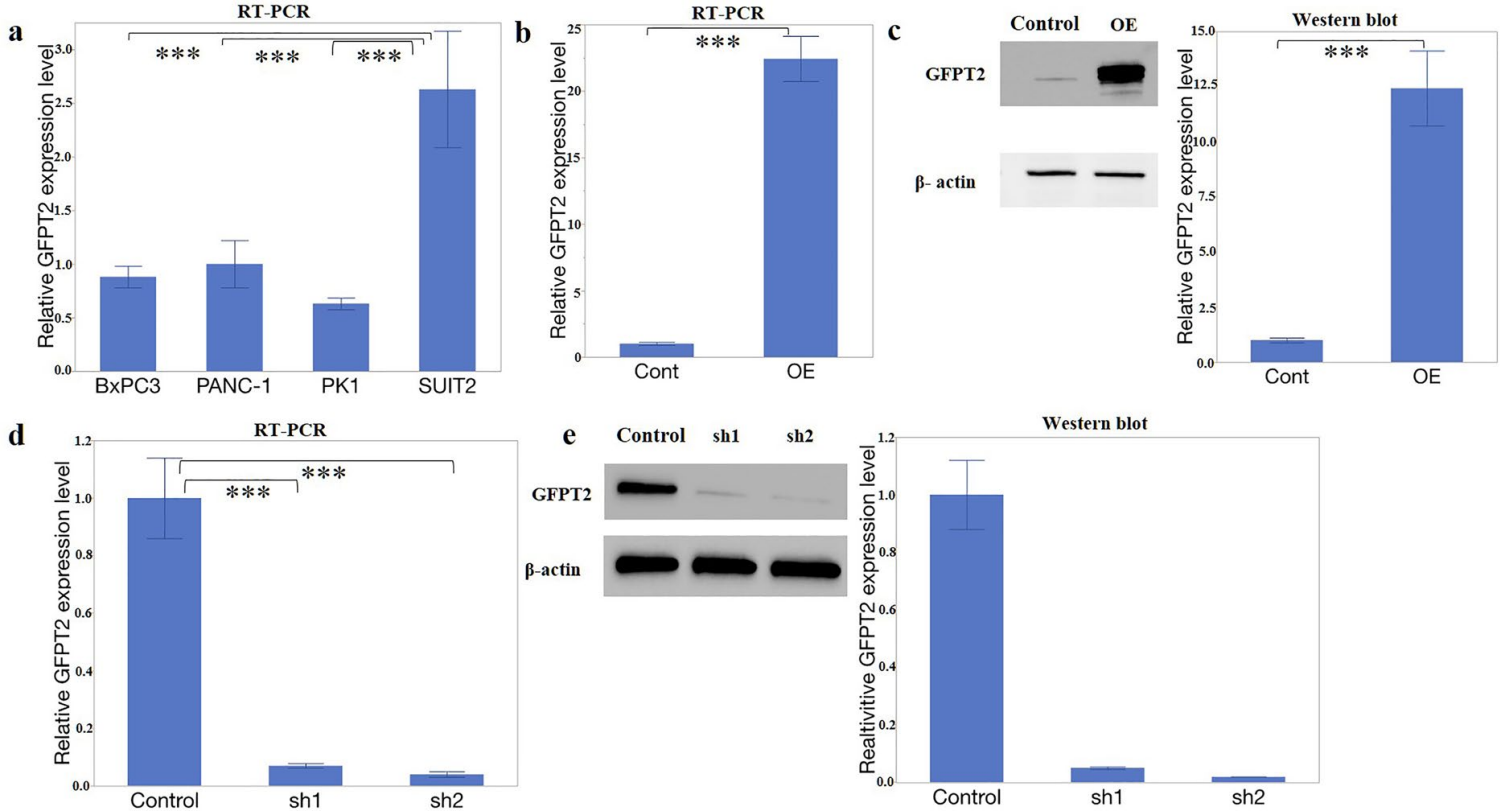
GFPT2 expression in PaCa cells. **a** *GFPT2* expression in PANC-1, SUIT2, BxPC3, and PK1 cells was evaluated via RT-qPCR. The relative expression level was calculated after normalization to the level of β-actin. **b** and **c** GFPT2 was overexpressed in PANC-1 cells. **b** RT-qPCR and **c** western blotting. (d and e) GFPT2 expression was suppressed by shRNA transfection in SUIT2 cells. **d** RT-qPCR and **e** western blotting. An empty vector was used as the control. β-Actin was used as an internal control. RT‑qPCR and western blotting were performed in a biological triplicate. *GFPT2* glutamine-fructose-6-phosphate transaminase 2; *PaCa* pancreatic cancer; *RT-qPCR* reverse transcription-quantitative polymerase chain reaction; sh, short hairpin

*GFPT2* expression levels were assessed using RT-qPCR in four PaCa cell lines: PANC-1, SUIT2, BxPC3, and PK1. GFPT2 expression was significantly elevated in SUIT2 cells, whereas no significant difference in GFPT2 expression was found among the BxPC3, PK1, and PANC-1 cells (Fig. 1a). Consequently, SUIT2 was chosen for the genotyping of stable GFPT2-suppressed cells, and PANC-1 was selected for the production of GFPT2-overexpressing cells, because these cells were used in our previous study [16].

After PANC-1 cells were transfected with the *GFPT2* expression vector, single-cell clones were obtained, and the expression levels of these genes increased 23.1-fold according to RT-qPCR (P < 0.001) and 12.5-fold according to WB (P < 0.001) (Fig. 1b, c). The designed shRNA was inserted into the pBAsi-hU6 NEO plasmid, which was subsequently transfected into SUIT2 cells. Following single-cell cloning, *GFPT2* expression decreased to 0.07-fold in SUIT2-sh1 cells (P < 0.001) and 0.04-fold in SUIT2-sh2 cells (P < 0.001), relative to that of the control cells, as determined using RT-qPCR (Fig. 1d). WB revealed that GFPT2 levels decreased to 0.05-fold in SUIT2-sh1 cells (P < 0.001) and 0.02-fold in SUIT2-sh2 cells (P < 0.001), relative to that in the control cells (Fig. 1e).

### GFPT2 regulates HBP activation in PaCa

**Fig. 2:**
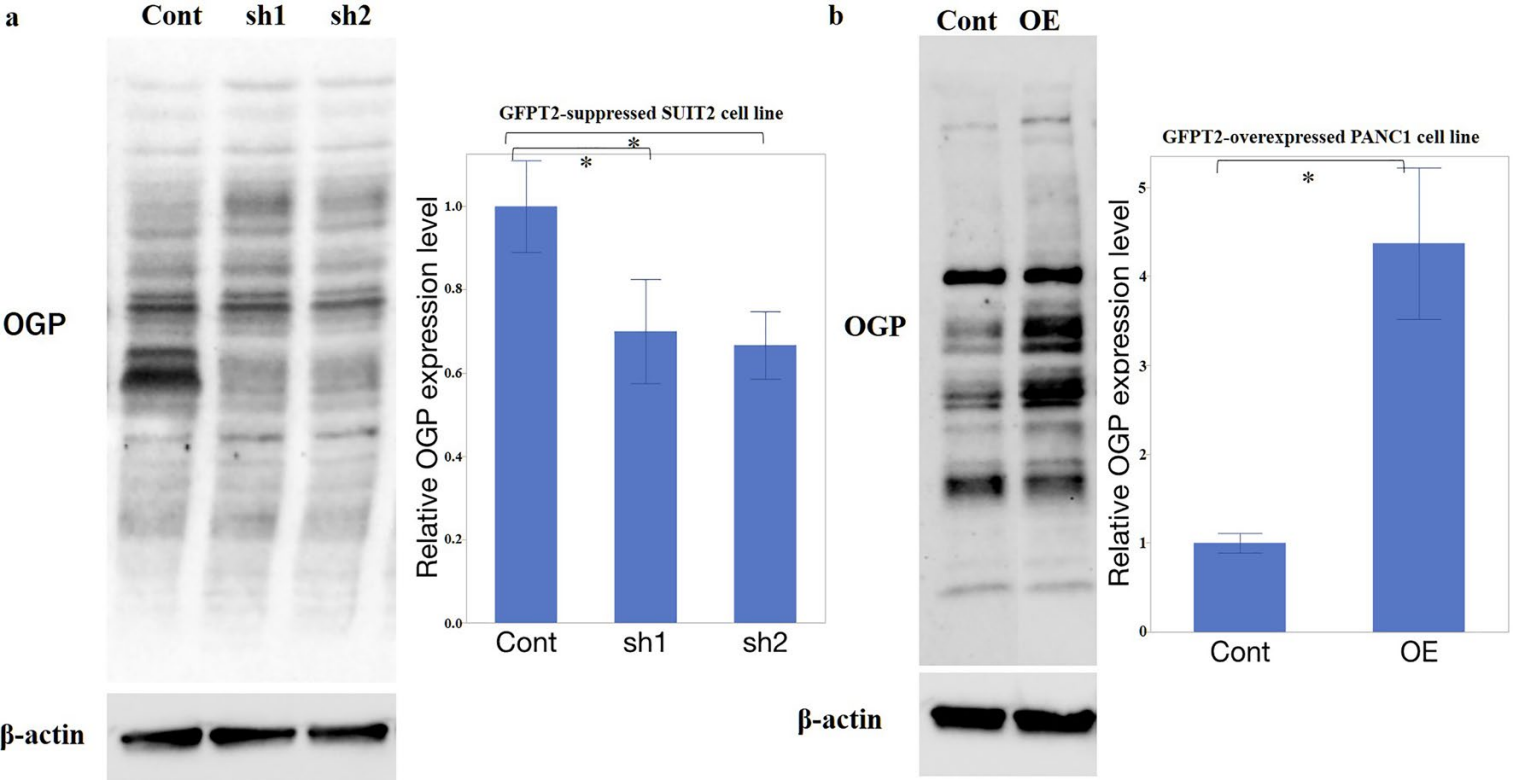
O-GlcNAc protein expression in PaCa cells **a** O-GlcNAc protein expression in GFPT2-suppressed SUIT2 cells. **b** O-GlcNAc protein expression in GFPT2-overexpressing PANC1 cells. β-Actin was used as an internal control. The data are expressed as the mean ± SD. All experiments were performed in a biological triplicate. *P < 0.05, **P < 0.01, and ***P < 0.001. *cont* control; *GFPT2* glutamine-fructose-6-phosphate transaminase 2; *O-Glc-Nac* O-N-acetylglucosamine; sh, short hairpin

To assess the effect of GFPT2 expression on HBP in PaCa cells, the expression of O-GlcNAc, the final product of HBP, was examined via WB. In GFPT2-suppressed SUIT2 cells, the overall expression of glycosylated proteins (O-GlcNAc protein: OGP) decreased to 0.70-fold in SUIT2-sh1 cells and 0.67-fold in SUIT2-sh2 cells, respectively (Fig. 2a). Conversely, the total expression of OGP increased to 4.18-fold in PANC1-OE cells (Fig. 2b). These findings suggest that, even in PaCa, GFPT2 expression may support HBP activation, regulating glycosylated protein levels.

### GFPT2 expression regulates migration and invasion through the HBP

**Fig. 3:**
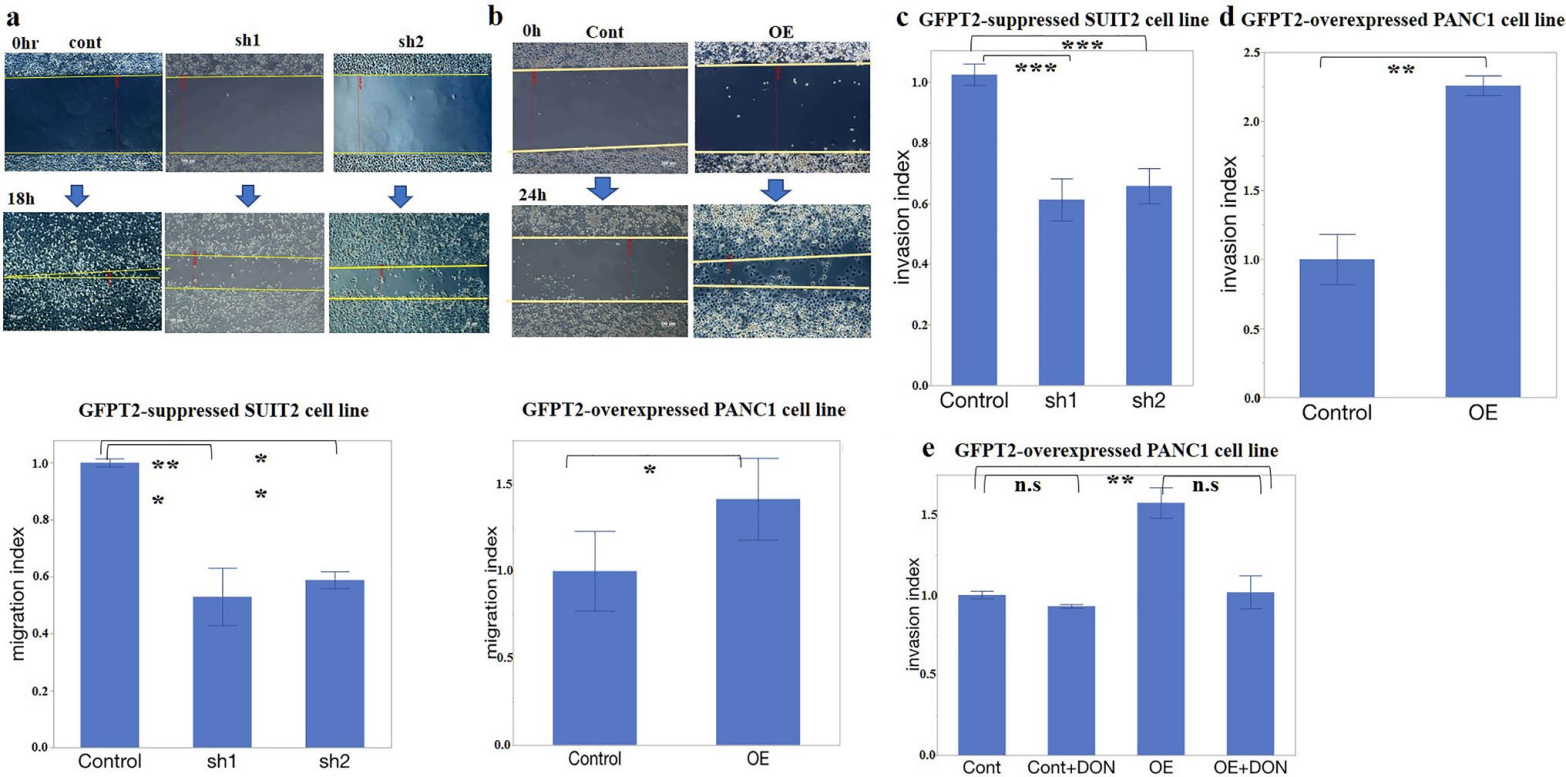
Migration of and invasion by PaCa cells. **a**, **b** Migration was assessed using a scratch assay. **a** Migrated cancer cells and the relative migration rate of GFPT2-suppressed SUIT2 cells. **b** Migrated cancer cells and the relative migration rate of GFPT2-overexpressing PANC-1 cells. Scale bar, 20 μm. **c**–**e** Transwell assays were performed to examine the association between cancer-cell invasion and GFPT2 expression. Invading cells were stained with lysis buffer/dye solution and quantified using a fluorescence plate reader with a 480/520 nm filter. **c** GFPT2-suppressed SUIT2 cells. **d** GFPT2-OE PANC-1 cells. **e** GFPT2-OE cells were treated with the HBP inhibitor, DON. The data are expressed as the mean ± SD. All experiments were performed in a biological triplicate. *P < 0.05, **P < 0.01, and ***P < 0.001. *GFPT2* glutamine-fructose-6-phosphate transaminase 2; cont, control; *DON* 6-diazo-5-oxo-L-norleucine; *GFPT2* glutamine-fructose-6-phosphate transaminase 2; *HBP* hexosamine biosynthetic pathway; *OE* overexpression; *sh* short hairpin

A wound-healing assay was conducted to assess the impact of GFPT2 on cell motility. The migration indices were significantly lower, at 0.54-fold (P < 0.001) for SUIT2-sh1 cells and 0.58-fold (P = 0.010) for SUIT2-sh2 cells (Fig. 3a). Conversely, in GFPT2-overexpressing cells, the migration index was 1.35-fold higher than that in PANC-1-OE cells (P = 0.031) (Fig. 3b).

Invasion activity was examined using a Boyden chamber coated with ECMatrix™. Similarly, compared to those of control cells, the invasion indices of SUIT-sh1 and SUIT-sh2 cells were 0.61-fold (P < 0.001) and 0.66-fold (P < 0.001), respectively (Fig. 3c). Among the GFPT2-overexpressing cells, the invasion index of PANC-1-OE cells was 2.26 times greater than that of control cells (Fig. 3d) (P = 0.003). The effects of DON, an HBP inhibitor, were evaluated to examine whether the increase in invasive activity caused by GFPT2 could be alleviated. DON, a glutamine analog that works as a glutaminase antagonist, was selected as it specifically competes with GFPT and inhibits metabolism of fructose-6-phosphate to glucosamine-6-phosphate. Exposing PANC1-OE cells to DON reduced the increase in invasive activity to the control levels (Fig. 3e). These findings suggest that GFPT2 may promote PaCa cell invasion by activating the HBP.

### GFPT2 does not affect cell proliferation or chemosensitivity to gemcitabine

**Fig. 4:**
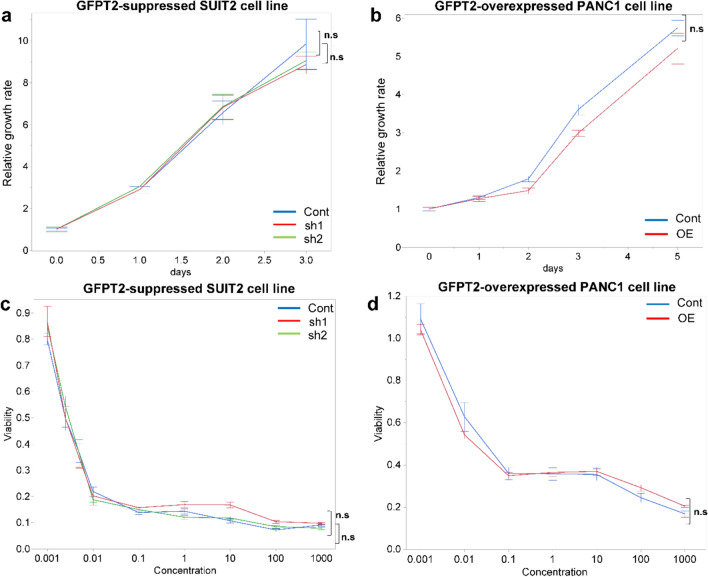
Proliferation and chemosensitivity of pancreatic cancer cells **a**, **b** Pancreatic cancer cell proliferation. **a** Proliferation curve for GFPT2-suppressed SUIT2 cells. **b** Proliferation curve for GFPT2-overexpressing PANC-1 cells. **c**, **d** Sensitivity of pancreatic cancer cells to gemcitabine. **c** IC_50_ did not differ among the cont, sh1, and sh2 groups for GFPT2-suppressed SUIT2 cells or **d** between the control and OE groups for GFPT2-overexpressing PANC-1 cells. All experiments were performed in biological triplicate. *Cont* control; *GFPT2* glutamine-fructose-6-phosphate transaminase 2; *OE* overexpression; *sh* short hairpin

The MTS assay results revealed nearly identical cell growth curves for both GFPT2-suppressed and GFPT2-overexpressing cells compared to those of the control cells. Moreover, no significant differences were found in the relative growth rates of GFPT2-suppressed cells on the third day (cont vs. sh1: P = 0.248; cont vs. sh2: P = 0.124) (Fig. 4a). Similar findings were obtained for the fifth-day growth rate of PANC-1-OE cells (P = 0.253) (Fig. 4b).

Cells were treated with various doses of gemcitabine to explore the effect of GFPT2 expression on chemosensitivity toward gemcitabine. The results indicated no significant differences in LD_50_ values between SUIT2-cont (0.003 µM), SUIT2-sh1 (0.003 µM), or SUIT2-sh2 (0.004 µM) cells (cont vs. sh1: P = 0.248; cont vs. sh2: P = 0.124) (Fig. 4c). Similarly, there were no significant differences in the LD_50_ values between PANC-1-cont cells (0.031 µM) and PANC-1-OE cells (0.013 µM; P = 0.150) (Fig. 4d). These results demonstrate that GFPT2 expression does not influence the proliferation or chemosensitivity of PaCa cells.

### GFPT2 induces EMT in pancreatic cancer cells

**Fig. 5:**
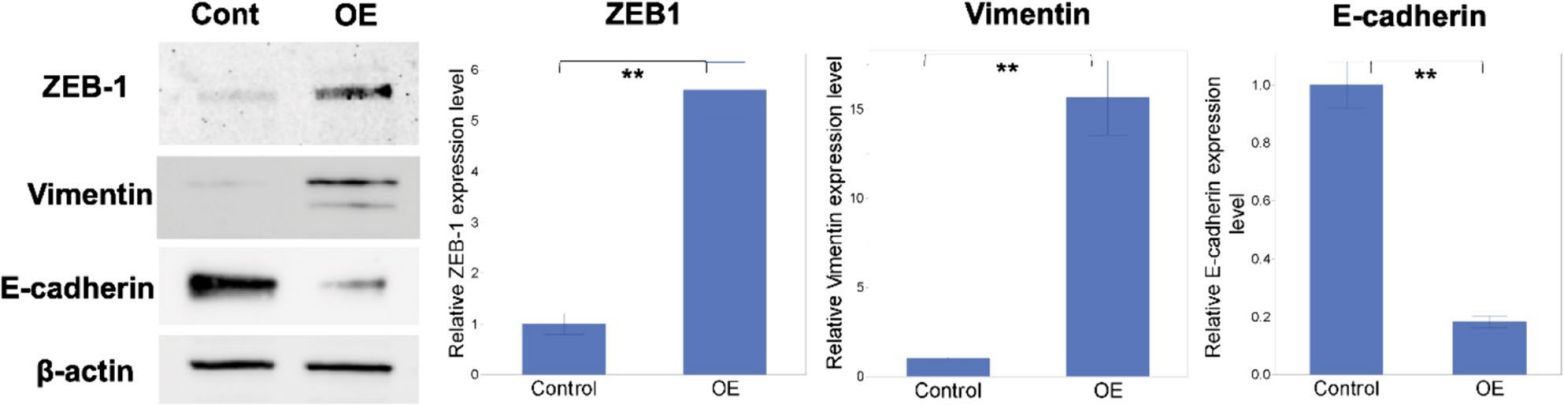
Expression of EMT-related proteins in PANC-1-OE cells. ZEB1 and vimentin expression was significantly elevated, while E-cadherin expression was significantly reduced. β-Actin was used as an internal control. The data are expressed as the mean ± SD. All experiments were performed in biological triplicate. *P < 0.05, **P < 0.01 and ***P < 0.001. *GFPT2* glutamine-fructose-6-phosphate transaminase 2; *OE* overexpression

GEPIA analysis of gene expression revealed a robust correlation between GFPT2 and ZEB1 and between GFPT2 and vimentin (Online Resource 3). Based on these findings, we assessed the expression of these factors in GFPT2-overexpressing PaCa cells. This revealed that elevated GFPT2 expression was associated with increased expression of ZEB1 and vimentin, along with a concomitant decrease in E-cadherin expression (Fig. 5). These findings suggest that the downstream signaling pathway involved in HBP activation is, in part, mediated by the induction of EMT.

### Orthotopically transplanted tumors exhibit increased expression of GFPT2, but not GFPT1, after GEM induction

**Fig. 6:**
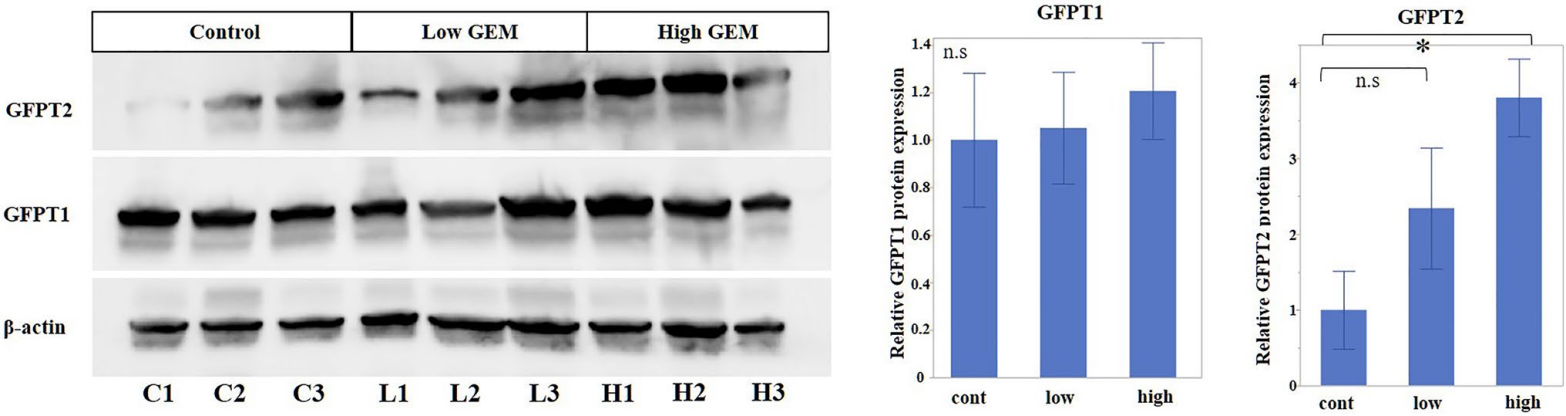
GFPT expression after gemcitabine administration in a mouse xenograft model. GFPT2 expression was significantly greater in the high-dose gemcitabine-treated group than in the control group. GFPT1 expression was not significantly altered after gemcitabine treatment. β-Actin was used as an internal control. Data are expressed as the mean ± SD. All experiments were performed in biological triplicate. *P < 0.05. GFPT1, glutamine-fructose-6-phosphate transaminase 1; *GFPT2* glutamine-fructose-6-phosphate transaminase 2; *GEM* gemcitabine

Gemcitabine administration was performed every week for 6 weeks following the confirmation of tumor formation, in an orthotopic mouse xenograft model. One week after the last induction, the tumors were excised, and the expression of GFPT1 and GFPT2 was assessed. The findings revealed a significant increase in GFPT2 expression in the high-dose GEM-treated group compared to that of the control group (Fig. 6). In contrast, GFPT1 expression did not differ significantly after GEM induction. These results indicate that the specific upregulation of GFPT2 expression was induced by GEM treatment.

### Neoadjuvant chemotherapy increases GFPT2 expression

**Fig. 7:**
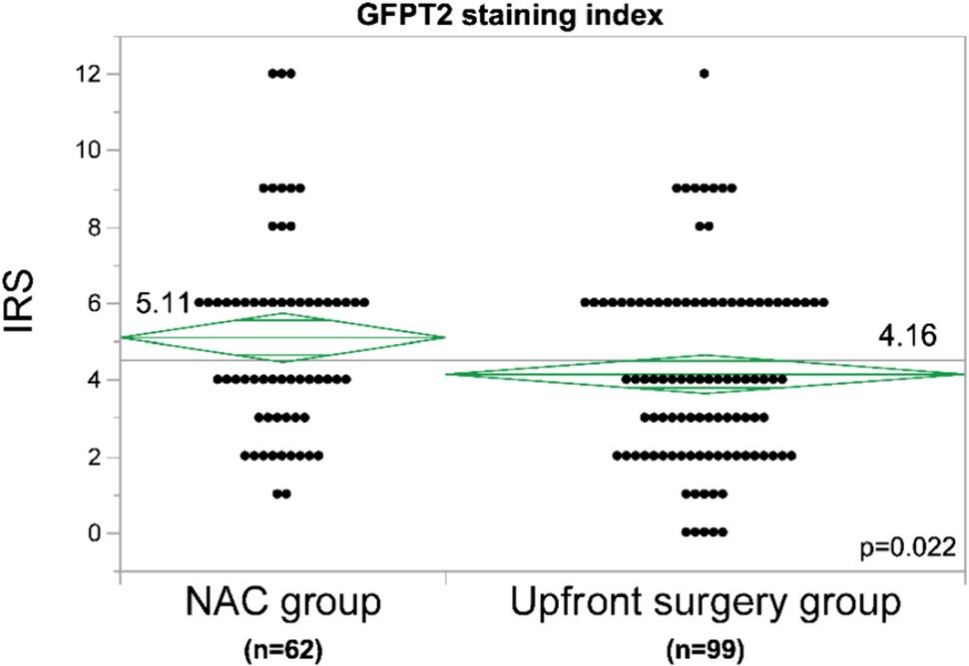
Comparison of GFPT2 expression between patients receiving NAC and upfront surgery. GFPT2 expression was significantly higher in patients undergoing NAC (P = 0.022). *GFPT2* glutamine-fructose-6-phosphate transaminase 2; *NAC* neoadjuvant chemotherapy; *IRS* immunoreactive score

Immunohistochemical analysis of GFPT2 expression in 161 resected PaCa patients revealed a significantly greater immunoreactivity score for GFPT2 in the neoadjuvant chemotherapy (NAC) group than in the upfront surgery group (IRS: 5.11 vs. 4.16, P = 0.022) (Fig. 7). The demographic characteristics of the neoadjuvant chemotherapy and upfront surgery groups are shown in Online Resource 4. Based on these findings, an optimal cut-off value of 5 was established; scores ≥ 5 indicated high expression, while those < 5 indicated low expression.

### GFPT2 expression is correlated with liver metastasis after GEM treatment

**Fig. 8:**
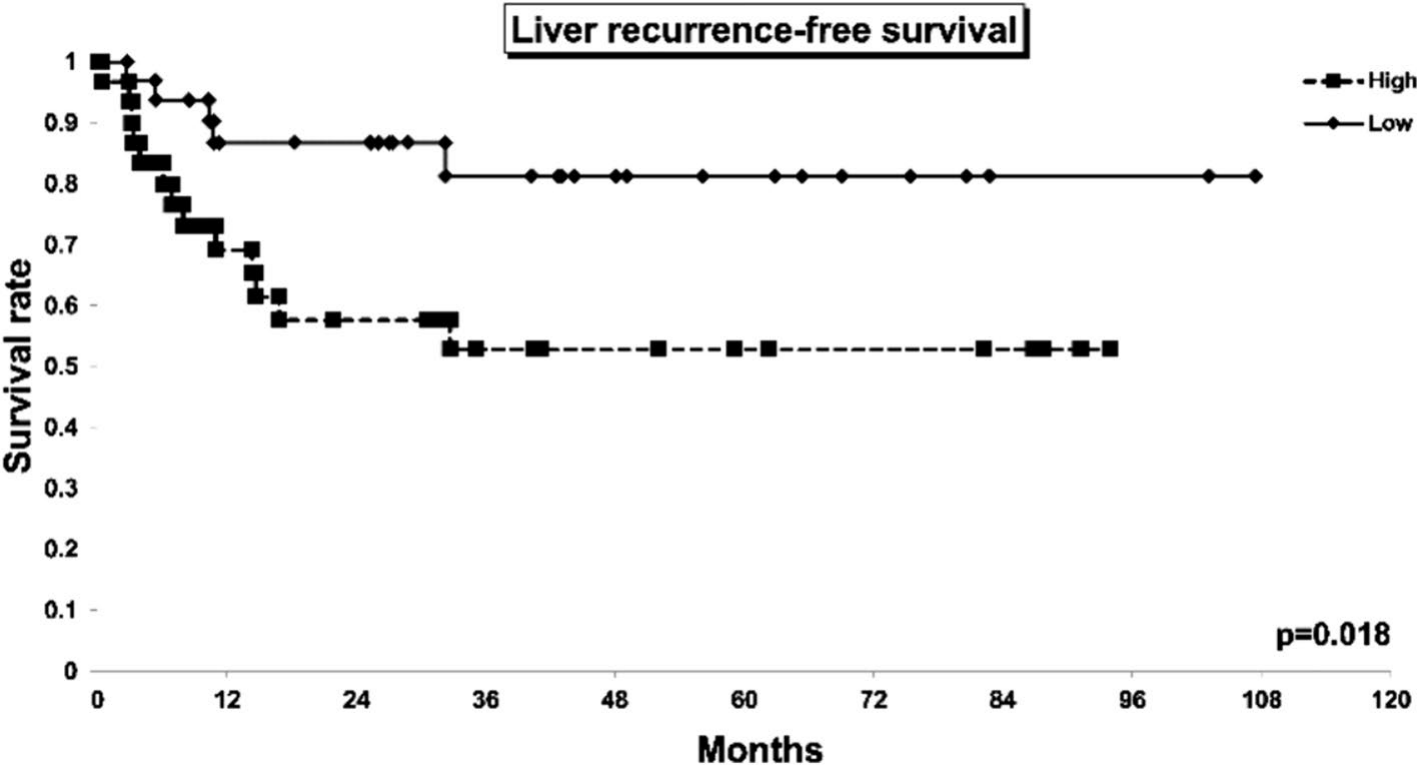
Kaplan–Meier curves of liver recurrence-free survival. Patients with high (n = 30) or low GFPT2 (n = 32) expression are represented by dots and lines, respectively. Liver recurrence: cancer recurrence occurring first in the liver following pancreatic cancer resection

The analysis focused on the clinical progression of 62 patients who received NAC and examined patient outcomes based on GFPT2 expression (Online Resource 5). Our findings revealed a greater incidence of cancer recurrence occurring first in the liver following PaCa resection in patients with elevated GFPT2 expression (n = 30) than in those with low GFPT2 expression (n = 32) (Fig. 8). Nevertheless, no statistically significant differences were observed in terms of overall survival or recurrence-free survival (Online Resource 6a, b). These findings indicate that GFPT2 expression is a potential predictor of liver recurrence after neoadjuvant chemotherapy.

## Discussion

This study revealed that GEM administration in a mouse xenograft model selectively increased the expression of GFPT2. GFPT2 expression was also upregulated after NAC and was strongly correlated with liver metastasis after surgical resection. Increased GFPT2 expression enhances invasion by activating the HBP and subsequently regulating EMT. These results suggested that GEM-induced GFPT2 expression plays an important role in promoting cancer cell metastasis.

The expression of GFPT2, not GFPT1, was selectively upregulated following GEM exposure. Several studies have shown that oxidative stress elevates the expression of GFPT2. In breast cancer, oxidative stress stimulation increases GFPT2 expression [32]. GFPT2 expression was also elevated under hypoxic conditions in genetically engineered mouse PaCa models. Under hypoxic conditions, GFPT1 expression increased 1.5-fold, whereas GFPT2 expression increased ninefold [33]. ROS stimulation increased GFPT2 expression [34]. These results from the literature suggest that GFPT2 expression is enhanced under hypoxia-induced oxidative stress or ROS stimulation. Anticancer drugs typically induce oxidative stress [35], and GEM increases intracellular ROS levels [15]. These findings from the literature suggest that stimulation by GEM may increase GFPT2 expression via ROS production. However, the detailed mechanism regulating GFPT2 expression, particularly downstream signaling of oxidative stress, has not been fully elucidated and still remains unclear.

This study revealed that GFPT2 expression promoted cancer cell invasion and that this increase in invasion was suppressed by DON, an HBP inhibitor. In our retrospective analysis of clinical samples, GFPT2 expression was also correlated with liver metastasis after NAC. These data suggest that GFPT2 plays an important role in promoting the metastatic potential of PaCa cells, by promoting cell invasion via HBP activation. Several studies have shown that HBP activation induces EMT by regulating the expression of EMT-related factors [21–23]. Furthermore, gene analysis using GEPIA revealed correlations between GFPT2 and several EMT-related factors. Therefore, the expression of EMT-related factors was evaluated in this study. The expression of ZEB1 and vimentin increased, whereas that of E-cadherin decreased. The transcription factor ZEB1 is involved in the suppression of E-cadherin expression [36] and has been reported to act as a transcription factor for vimentin [37, 38]. Taken together, GFPT2-induced HBP activation may induce EMT by regulating ZEB1 expression.

The mechanism underlying the EMT caused by HBP activation has been revealed in several cancers. GlcNAc is the final metabolite of HBP and activates several cancer-related factors through post-transcriptional modifications including glycosylation. The activation of several factors, such as β-catenin [21], c-Myc [22], and NFκB [23] regulates the expression of EMT-related factors. In colorectal cancer, HBP activation promotes NFκB activation via glycosylation and induces EMT [23]. In serous ovarian cancer, β-catenin glycosylation induces nuclear translocation and promotes cell migration and invasion through EMT [21]. Furthermore, in breast cancer, GFPT2 induces EMT by increasing vimentin expression via HBP activation and enhances cell invasion and proliferation [32]. However, there are no reports concerning PaCa, and the mechanism linking HBP activation to EMT has not yet been elucidated. The results of this study demonstrated that increased GFPT2 expression elevated the total expression level of GlcNAc, indicating that HBP was activated and the glycosylation of some factors was induced. Although the target factor was not elucidated in this study, the results suggest that GFPT2-induced glycosylation might regulate the expression level of ZEB1 by activating transcription factors.

In PaCa, GFPT1 plays an important role in tumor progression. GFPT1 expression induces HBP activation and promotes malignancy through the glycosylation of β-catenin [25]. High GFPT1 expression is also associated with poor prognosis in PaCa [26, 39]. In contrast, GFPT2 has not been recognized as an important contributing factor to PaCa malignancy. This study highlights the role of GFPT2 as its expression is selectively increased under specific conditions. Furthermore, increased GFPT2 activated the HBP, similar to what has been observed for GFPT1 in PaCa. These mechanisms indicated that, under particular conditions, HBP was activated by increasing the expression of GFPT2, another phenotype of GFPT1. However, whether increased GFPT2 expression causes further HBP activation and leads to EMT in all PaCa cells is unclear because HBP might already be activated to some extent by GFPT1. Therefore, whether this effect is limited to patients with low GFPT1 expression or to all PaCa patients needs to be elucidated in future studies.

Chemotherapy can lead to EMT and alter the tumor microenvironment, thus promoting metastasis [6, 40]. Both the tumor and its microenvironment should be considered in CIM. Hashin et al. [40] demonstrated that, while low-dose GEM inhibits the incidence of metastasis, high-dose GEM alters the host-derived microenvironment and induces myeloid-derived suppressor cells, which promote metastasis. In mice, high-dose GEM treatment suppressed orthotopic-transfected tumor growth, whereas low-dose GEM treatment promoted metastasis [11]. This process may be mediated by CIM in the tumor microenvironment. Low-dose GEM is unable to suppress cancer cell growth, and continuous stimulation was added in the surviving cells. Our findings suggest that GEM enhances GFPT2 expression in the PaCa cells. Furthermore, GFPT2 expression activates HBP and enhances invasion via EMT. These results suggest that continuous GEM stimulation causes the surviving cells to acquire metastatic potential by regulating metabolic pathways, potentially reflecting CIM. Considering that GEM administration induces various changes in the tumor microenvironment, further research on CIM should consider both the tumor and its microenvironment.

Several drugs have been developed to inhibit the HBP. In the present study, DON suppressed GFPT2-induced cell invasion, suggesting that an HBP inhibitor may be an effective therapeutic tool for preventing GFPT2-induced metastasis. DON is one of the oldest HBP inhibitors. In the 1950s and the 1960s, clinical trials of DON were conducted for various cancers, such as leukemia, breast cancer, and lung cancer [41]. However, these approaches do not lead to favorable outcomes, and several side effects due to mucosal damage prevent its further use in clinical practice. In the 1980s–2000s, phase II trials were conducted on lung and colorectal cancers [41], but the efficacy of these agents remained poor. Considering these results, DON may not be a favorable treatment option when used as a single agent. The present study suggests that GEM increases GFPT2 expression and that GFPT2 promotes HBP activation. Furthermore, DON prevented GFPT2-induced invasion. These data suggest that combination therapy with anticancer agents may be effective for preventing metastasis. Considering the results of several underlying clinical trials, and its well-established safety profile, clinical trials could be easily initiated if the mechanisms of CIM and the effects of HBP become clearer. Further studies are required to investigate the potential effects of DON in combination with other anticancer drugs.

This study has several limitations. First, this study did not directly evaluate the mechanism of chemotherapy-induced migration or invasion; thus, it is unclear whether the inhibition of HBP could directly prevent metastasis caused by chemotherapy. However, several studies have investigated chemotherapy-induced EMT and CIM [6, 42, 43]. Therefore, chemotherapy-induced elevation of GFPT2 expression is thought to be strongly correlated with this mechanism. For future studies, establishing GEM resistant PaCa cells may serve as a good model for evaluating the relationship between GEM-induced GFPT2 expression and GFPT2-induced invasion activity, as these cells have been reported to be induced by EMT and increased invasion activity [42, 43]. Second, there were several biases in the immunohistochemical analysis, as the preoperative chemotherapy group had more advanced tumors (Online Resource 7). This bias suggests that elevated GFPT2 expression was caused not only by neoadjuvant chemotherapy but also by tumor progression. Third, this study used only one anticancer drug, GEM, for stimulation. Thus, whether HBP activation can be induced by other anticancer drugs has not been thoroughly investigated. Finally, we used only one HBP inhibitor in this study. Although there are several HBP inhibitors, including FR054 [44], this study used DON because it selectively and functionally competes with GFPT2 and is suitable for analyzing the effect of GFPT2. However, other agents may be more effective for inhibiting HBP activation and invasive activity. Although there are several limitations, this is the first study to show that GEM stimulation selectively increases GFPT2 expression in PaCa cells and that increased GFPT2 promotes invasive activity via HBP activation. Chemotherapy is the key treatment for PaCa and is expected to prolong patient survival. However, a recent study has revealed that the chemotherapy itself induce metastasis, called CIM. GEM-induced GFPT2 expression indicated that GEM could potentially enhance metastatic ability via HBP activation. In the future, the detailed mechanisms of CIM and HBP should be elucidated to prevent undesired effects of chemotherapy.

## Conclusions

This study revealed that GEM stimulation selectively increases the expression of GFPT2, a rate-limiting enzyme of the HBP. GFPT2 expression activates HBP and promotes PaCa cell invasion. The analysis of clinical samples show that chemotherapy increases the expression of GFPT2, and that liver recurrence is frequent in GFPT2-expressing patients. Together, these results suggest that GEM-induced GFPT2 expression has an important role in promoting PaCa cell metastasis.

## Supplementary Information

Below is the link to the electronic supplementary material.

Supplementary file1 (DOCX 733 KB)

## Data Availability

The datasets used and/or analyzed in the current study are available from the corresponding author upon reasonable request.
