# Supplementary material for: GFPT2 expression is induced by gemcitabine administration and enhances invasion by activating the hexosamine biosynthetic pathway in pancreatic cancer
Source: Clin Exp Metastasis. 2024 Jun 18;41(5):777–89. doi: 10.1007/s10585-024-10298-y (PMC11499537; doi:10.1007/s10585-024-10298-y)
Supplement: Supplementary file 1 — Supplementary file1 (DOCX 733 KB) [file 10585_2024_10298_MOESM1_ESM.docx]

**Supplementary Materials**

*Clinical & Experimental Metastasis*

**GFPT2 expression is induced by gemcitabine administration and enhances invasion by activating the hexosamine biosynthetic pathway in pancreatic cancer**

Kent Miyazaki^1^, Kyohei Ariake^1,2^*, Satoko Sato^3^, Takayuki Miura^1^, Xun Jing Yu^1^, Daisuke Douchi^1^, Masaharu Ishida^1^, Hideo Ohtsuka^1^, Masamichi Mizuma^1^, Kei Nakagawa^1^, Takashi Kamei^1^, and Michiaki Unno^1^

^1^Department of Surgery, Tohoku University Graduate School of Medicine, Sendai, Japan

^2^Department of Gastroenterological Surgery, Sendai City Medical Center Sendai Open Hospital, Sendai, Japan

^3^Department of Pathology, Tohoku University Hospital, Sendai, Japan

***Corresponding author**: Kyohei Ariake, E-mail: [ariake@surg.med.tohoku.ac.jp](mailto:ariake@surg.med.tohoku.ac.jp)

**Online Resource 1** shRNA and primer sequences used in this study

| shRNA category | Sequence |
| --- | --- |
| shRNA1 | Top:5′GATCCCAAGTTTGCGTATAAGACACTGTGAAGCCACAGATGGGTGTCTTATACGCAAACTTGTTTTTTA3′  Bottom:5′AGCTTAAAAAACAAGTTTGCGTATAAGACACCCATCTGTGGCTTCACAGTGTCTTATACGCAAACTTGG3′ |
| shRNA2 | Top:5′GATCCGAATAATCACGAAGTCAAACTGTGAAGCCACAGATGGGTTTGACTTCGTGATTATTCTTTTTTA3′  Bottom:5′AGCTTAAAAAAGAATAATCACGAAGTCAAACCCATCTGTGGCTTCACAGTTTGACTTCGTGATTATTCG3′ |

| **Primer category** | **Sequence** |
| --- | --- |
| GFPT2 | Forward:5′-GCTCATCGTGATTGGCTGTGGA-3′  Reverse:5′-CAACCATCACAGGAAGCTCAGTC-3′ |
| β-actin | Forward:5′-CACCATTGGCAATGAGCGGTTC-3′  Reverse:5′-AGGTCTTTGCGGATGTCCACGT-3′ |


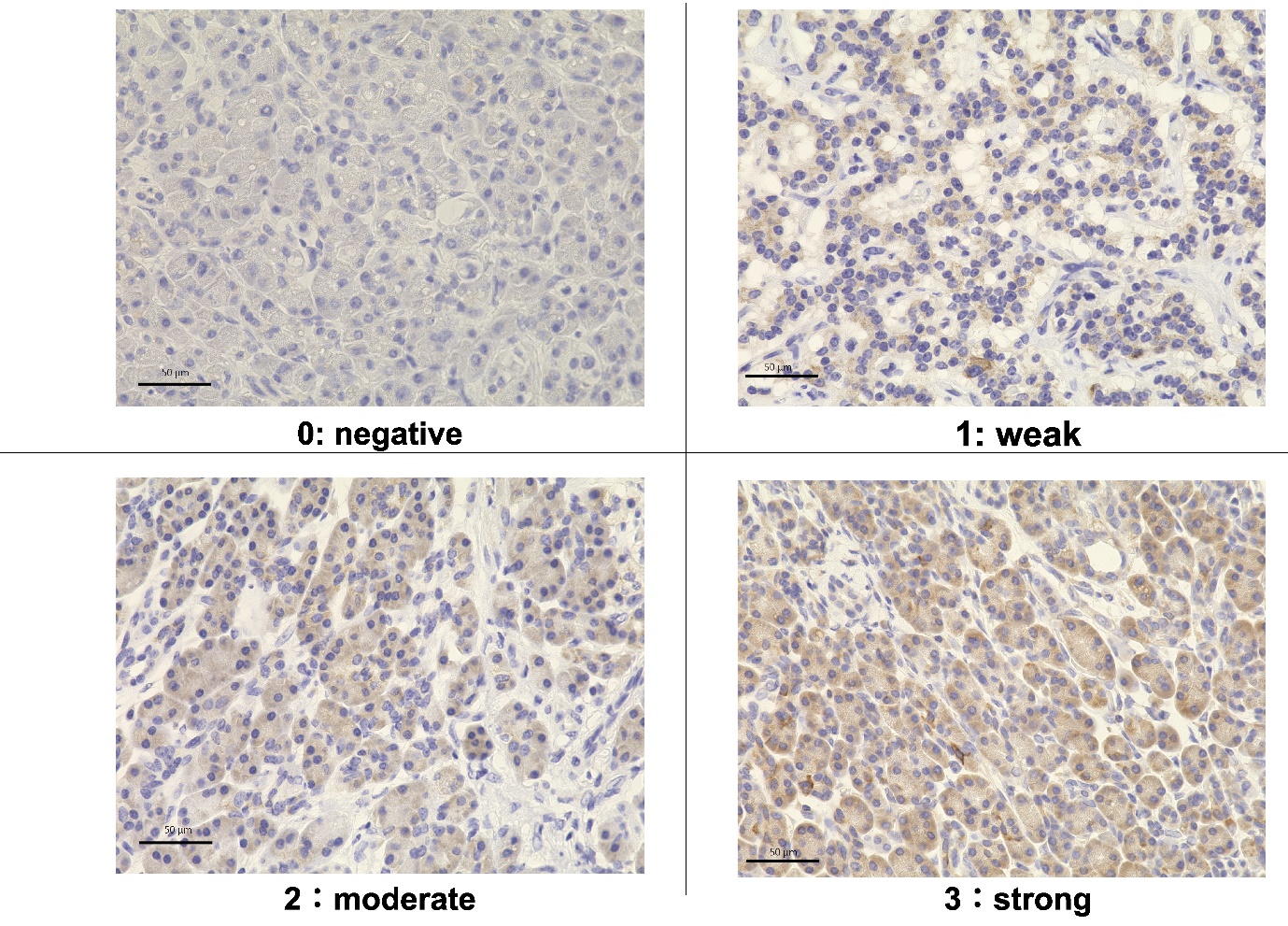


**Online Resource 2** Immunohistochemical staining for GFPT2 in pancreatic cancer

Staining intensity is scored as (a) 0 (negative), (b) 1 (weak), (c) 2 (moderate), or (d) 3 (strong). GFPT2, glutamine-fructose-6-phosphate transaminase

**

**

**Online Resource 3** Gene analysis using GEPIA

(a) Correlation between GFPT2 and ZEB1 expression in patients with pancreatic cancer from large-scale RNA sequence data from GEPIA. (b) Correlation between GFPT2 and ZEB1 vimentin expression. These data show a strong correlation between GFPT2, vimentin, and ZEB1 expression

**Online Resource 4** Clinical characteristics of patients

|  | | **Neoadjuvant chemotherapy**  **(n = 62)** | **Upfront surgery**  **(n = 99)** | **P-value** |
| --- | --- | --- | --- | --- |
| Age (year) | Median | 66 (41–80) | 70 (47–88) | 0.041 |
| Sex | Male:Female | 28:34 | 27:20 | 0.733 |
| Resectability | R:BR:UR | 19:36:7 | 71:27:1 | <0.001 |
| Pretreatment CA19-9 | Median | 564 | 571.1 | 0.972 |
| Residual cancer | R0:R1 | 57:5 | 85:14 | 0.832 |
| Adjuvant chemotherapy | Yes:No | 57:5 | 76:23 | 0.018 |
| Tumor position | Ph:Pbt | 43:19 | 62:37 | 0.493 |
| Tumor size (mm) | median | 26.8 | 27.6 | 0.659 |
| Artery invasion | No:Yes | 56:6 | 95:4 | 0.185 |
| Portal vein invasion | No:Yes | 33:29 | 69:30 | 0.044 |
| UICC-T | 1+2:3 | 4:58 | 10:89 | 0.569 |
| UICC-N | 0:1 | 22:40 | 31:68 | 0.608 |
| GFPT2 staining index | Average | 5.11 | 4.16 | 0.022 |

**Online Resource 5** Clinical characteristics of patients who received neoadjuvant chemotherapy

|  | | **Neoadjuvant chemotherapy**  **(n = 30)** | **Upfront surgery**  **(n = 32)** | **P-value** |
| --- | --- | --- | --- | --- |
| Age (year) | Median | 65.5 (50–78) | 67 (41–80) | 0.887 |
| Sex | Male:Female | 17:13 | 17:15 | 0.779 |
| Resectability | R:BR:UR | 7:18:5 | 12:18:2 | 0.281 |
| Pretreatment CA19-9 | Median | 113.3 (2–1134100) | 193.8 (0.6–5154) | 0.578 |
| Residual cancer | R0:R1 | 27:3 | 30:2 | 0.588 |
| Adjuvant chemotherapy | Yes:No | 24:6 | 28:4 | 0.422 |
| Tumor position | Ph:Pbt | 22:8 | 20:12 | 0.362 |
| Tumor size (mm) | median | 28.5 (10–44) | 25 (11–50) | 0.154 |
| Artery invasion | No:Yes | 27:3 | 29:3 | 0.934 |
| Portal vein invasion | No:Yes | 15:15 | 18:14 | 0.622 |
| UICC-T | 1+2:3 | 1:29 | 3:29 | 0.333 |
| UICC-N | 0:1 | 9:21 | 13:19 | 0.382 |





**Online Resource 6** Kaplan–Meier curves for overall survival and recurrence-free survival

(a) Overall survival and (b) recurrence-free survival. High (n = 30) or low GFPT2 (n = 32) expression levels are represented using dots or lines, respectively

**Online Resource 7** Clinical characteristics of patients with high or low GFPT2 expression

|  | | **GFPT2 expression** | | **P-value** |
| --- | --- | --- | --- | --- |
|  |  | **high**  **(n = 67)** | **low**  **(n = 94)** |  |
| Age (year) | Median | 67 (50–85) | 69 (41–88) | 0.810 |
| Sex | Male:Female | 39:28 | 52:42 | 0.715 |
| Resectability | R:BR:UR | 30 33:4 | 60:31:3 | 0.054 |
| Pretreatment CA19-9 | Median | 150 | 94.4 | 0.116 |
| Residual cancer | R0:R1 | 56:11 | 86:8 | 0.125 |
| Adjuvant chemotherapy | Yes:No | 54:13 | 79:15 | 0.571 |
| Neoadjuvant chemotherapy | Yes:No | 30:37 | 32:62 | 0.224 |
| Tumor position | Ph:Pbt | 52:15 | 52:42 | 0.003 |
| Tumor size (mm) | median | 30 | 25 | >0.001 |
| Artery invasion | No:Yes | 62:5 | 88:6 | 0.790 |
| Portal vein invasion | No:Yes | 37:30 | 65:29 | 0.044 |
| UICC-T | 1+2:3 | 1:66 | 13:81 | 0.014 |
| UICC-N | 0:1 | 16:51 | 37:57 | 0.058 |
